# Impact of Lumbar Surgery on Pharmacological Treatment for Patients with Lumbar Spinal Canal Stenosis: A Single-Center Retrospective Study

**DOI:** 10.3390/jcm12062385

**Published:** 2023-03-20

**Authors:** Takaya Imai, Sota Nagai, Takehiro Michikawa, Risa Inagaki, Soya Kawabata, Kaori Ito, Kurenai Hachiya, Hiroki Takeda, Daiki Ikeda, Shigeki Yamada, Nobuyuki Fujita, Shinjiro Kaneko

**Affiliations:** 1 Department of Orthopaedic Surgery, School of Medicine, Fujita Health University, Toyoake 470-1192, Japan; 2 Department of Spine and Spinal Cord Surgery, Fujita Health University, Toyoake 470-1192, Japan; 3 Department of Environmental and Occupational Health, School of Medicine, Toho University, Tokyo 143-8541, Japan; 4 Department of Clinical Pharmacy, School of Medicine, Fujita Health University, Toyoake 470-1192, Japan; 5 Department of Hematology, School of Medicine, Fujita Health University, Toyoake 470-1192, Japan

**Keywords:** lumbar spinal canal stenosis, lumbar surgery, pharmacological treatment, Roland-Morris Disability Questionnaire, Zurich Claudication Questionnaire, Japanese Orthopaedic Association Back Pain Evaluation Questionnaire, surgical effectiveness, walking ability, social life

## Abstract

Treatment for lumbar spinal canal stenosis (LSCS) is mainly classified into conservative and surgical therapies. Among conservative therapies, pharmacological treatment is commonly prescribed for LSCS. Meanwhile, surgical treatment is the last option for LSCS. This study aimed to examine the impact of lumbar surgery on pharmacological treatment for patients with LSCS. Consecutive patients aged ≥ 40 years who underwent lumbar surgery for LSCS were identified. A total of 142 patients were retrospectively reviewed for preoperative and 6-month and 1-year postoperative LSCS medications. The results showed that the number of LSCS medications significantly decreased after lumbar surgery. The proportion of the patients taking non-steroidal anti-inflammatory drugs, pregabalin/mirogabalin, opioids, prostaglandin E1 analogs, and neurotropin was significantly decreased after lumbar surgery, but that of the patients taking mecobalamin, acetaminophen, and serotonin-noradrenalin reuptake inhibitors was not significantly changed. Additionally, around 15% of the participants showed an increase in LSCS medications even after lumbar surgery. Multivariable analysis revealed that individuals without improvements in walking ability (RR: 2.7, 95% CI: 1.3–5.9) or social life (RR: 2.3, 95% CI: 1.1–5.0) had a greater risk of a postoperative increase in LSCS medications. The study results may provide physicians with beneficial information on treatment for LSCS.

## 1. Introduction

Lumbar spinal canal stenosis (LSCS) is a syndrome in which the spinal canal or foramen of the lumbar region is narrowed by bony, intervertebral, or ligamentous elements, resulting in cauda equina or nerve root compression. Patients with LSCS develop numbness and pain in the lower extremities and intermittent claudication as distinctive clinical manifestations. Intermittent claudication due to LSCS is clinically classified into three types: the cauda equina type, nerve root type, and combined type [1]. Additionally, LSCS is divided into congenital and acquired etiologies. The latter can be paraphrased as degenerative and accounts for most LSCS cases [2]. In the United States, the prevalence of LSCS was reported to be about 7% overall and increases with age [3]. In Japan, the prevalence of symptomatic LSCS is approximately 9%, and the increasing prevalence with age was reported to differ by gender [4]. With the global aging population, the prevalence of LSCS is expected to increase further [5].

LSCS treatment is mainly classified into conservative and surgical therapies [1,2]. Conservative therapy includes pharmacological treatment, physical therapy/exercise, spinal injection, manipulation, and ancillary treatments, such as bracing, traction, and electrical stimulation [1,2]. Among these treatments, pharmacological treatment is commonly prescribed for LSCS. It includes many medication options: non-steroidal anti-inflammatory drugs (NSAIDs), opioids, serotonin-noradrenalin reuptake inhibitors (SNRIs), pregabalin/mirogabalin, prostaglandin E1 analogs (PGE1), acetaminophen, mecobalamin, and neurotropin [1,2,6]. Meanwhile, surgical treatment is the last option for patients with LSCS, considering that it is indicated for patients who are resistant to conservative treatments including pharmacological treatment [1,2]. Surgical treatment is mainly divided into decompression alone and decompression with lumbar fusion. The former has favorable outcomes for patients without lumbar instability, and the latter has favorable outcomes for patients with lumbar instability [1]. Although patients who underwent lumbar surgery should be less dependent on conservative treatment, even patients after lumbar surgery occasionally show an increase in the intensity of conservative therapies. Pharmacological treatment is no exception, so postoperative patients with LSCS would theoretically have fewer LSCS medications. However, to the best of our knowledge, no studies have examined in detail the changes in LSCS medications before and after lumbar surgery for patients with LSCS.

Thus, the primary objective of this study was to examine the impact of lumbar surgery on pharmacological treatment for patients with LSCS. The secondary objective was to identify factors associated with patients with LSCS who experience an increase in LSCS medications after surgery.

## 2. Materials and Methods

### 2.1. Study Participants

The study design was a retrospective study. All the consecutive patients aged ≥ 40 years who underwent lumbar surgery for LSCS at our institution between April 2020 and March 2021 were identified, and retrospectively reviewed for preoperative and 6-month and 1-year postoperative information. The diagnosis and surgical indication for LSCS followed the guidelines [1,2]. Lumbar surgery was performed by six experienced spinal surgeons. Spondylolisthesis and degenerative lumbar scoliosis were diagnosed according to a previous study [7]. The case that had previously undergone lumbar surgery at the same level was defined as failed back surgery syndrome (FBSS). In surgical treatment, lumbar fusion was basically indicated for patients with LSCS with degenerative spondylolisthesis, degenerative lumbar scoliosis, and FBSS.

### 2.2. Ethics Approval

This study was approved by the Ethics Committee of Fujita Health University and conducted in accordance with the principles of the Declaration of Helsinki. Informed consent was obtained in the form of an opt-out on the website.

### 2.3. Data Collection

In the present study, pharmacists met the patients face to face at the outpatient department to check and record their medications preoperatively and 6-month and 1-year postoperatively. Simultaneously, the Roland-Morris Disability Questionnaire (RDQ), Zurich Claudication Questionnaire (ZCQ), and Japanese Orthopaedic Association Back Pain Evaluation Questionnaire (JOABPEQ) were assessed for the patients. Data on age, gender, body mass index (BMI), medical history including type 2 diabetes mellitus, hypertension, dyslipidemia, cardiovascular disease, cerebrovascular disease, cancer, spondylolisthesis, degenerative lumbar scoliosis, FBSS, decompression with or without fusion, surgical time, surgical blood loss, and incidence of dural tear were retrospectively collected from the patients’ medical records. The effectiveness of treatment in JOABPEQ was evaluated according to a previous study: a postoperative score increase of 20 points or more from the preoperative score, or a preoperative score of less than 90 and a postoperative score of 90 points or more [8]. A portion of the data has been used in our other study [9].

### 2.4. Statistical Analyses

The data among groups were compared using the chi-square test, McNemar test, or Wilcoxon signed-rank test, as appropriate. *p* values < 0.05 were considered to indicate statistical significance. When the McNamer and Wilcoxon signed-rank tests were used more than once, a *p* value of 0.025 was used as a statistical significance. We used a Poisson regression model that included age, sex, BMI, history of diabetes, with or without fixation, incidence of dural tear, and estimated relative risk (RR) and 95% confidence intervals (CIs) for the 1-year postoperative increase in LSCS medications to explore the factors associated with a 1-year postoperative increase in LSCS medications. Poisson regression was performed using the STATA16 software (Stata Corporation, College Station, TX, USA).

## 3. Results

**Figure 1:**
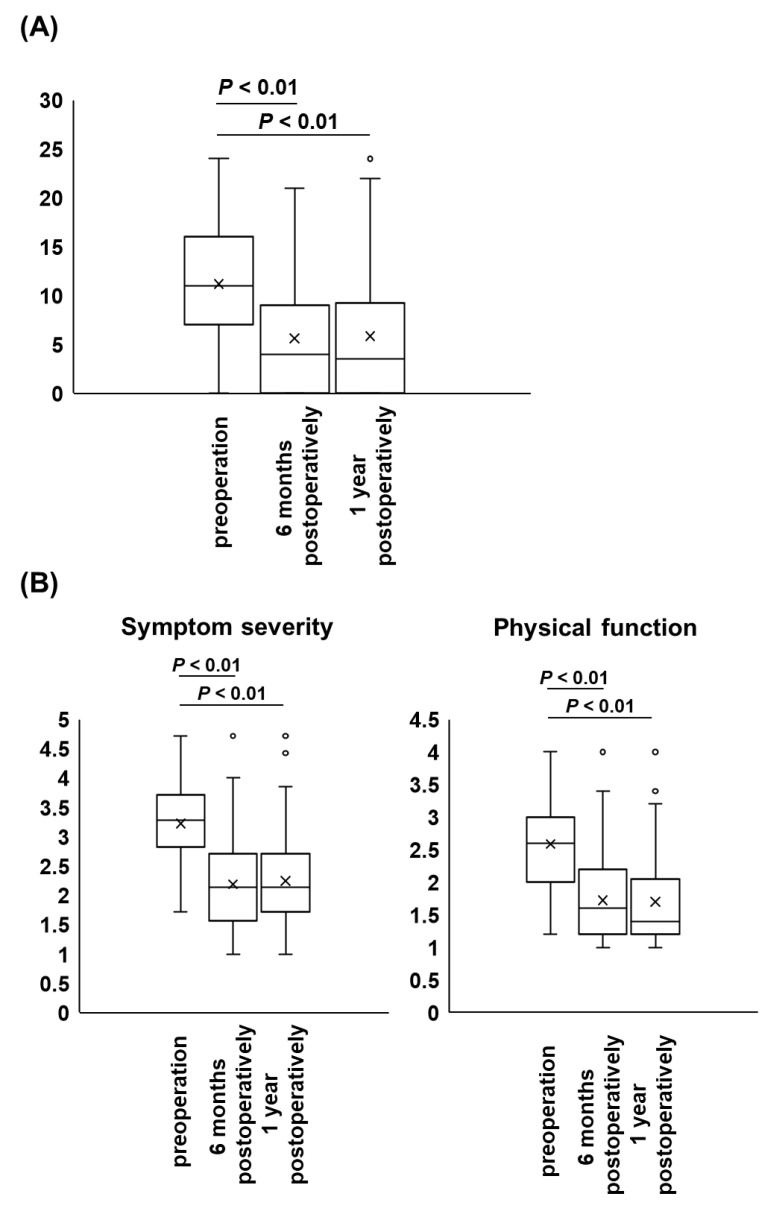
Box-and-whisker plot for comparison of valuables of patient-reported outcome at baseline and follow-up after surgery (*n* = 142). Both 6-month and 1-year postoperative scores of Roland-Morris Disability Questionnaire (**A**) and symptom severity ((**B**), left panel) and physical function ((**B**), right panel) of Zurich Claudication Questionnaire significantly improved compared to the preoperative scores.

A total of 142 patients were retrospectively reviewed in this study. One case with an additional lumbar surgery for epidural hematoma the day after initial surgery was included. The baseline characteristics of the participants are summarized in Table 1. Spondylolisthesis was diagnosed in 35.2% of cases, degenerative lumbar scoliosis in 11.2%, and FBSS in 7.7% (Table 1). In surgical treatment, decompression alone was performed in 59.2% of cases, and decompression with lumbar fusion was performed in the remaining 40.8% (Table 1). An incidental dural tear during lumbar surgery occurred in 9.2% of cases (Table 1). Figure 1 shows preoperative and 6-month and 1-year postoperative scores of RDQ and ZCQ. The results showed that both the 6-month and 1-year postoperative scores of RDQ and symptom severity and physical function of ZCQ significantly improved compared to the preoperative scores. Table 2 shows the number of effective cases of surgical treatment on JOABPEQ. The frequency of effective cases in each domain of JOABPEQ was comparable to that of previous reports (Table 2) [10,11].

**Figure 2:**
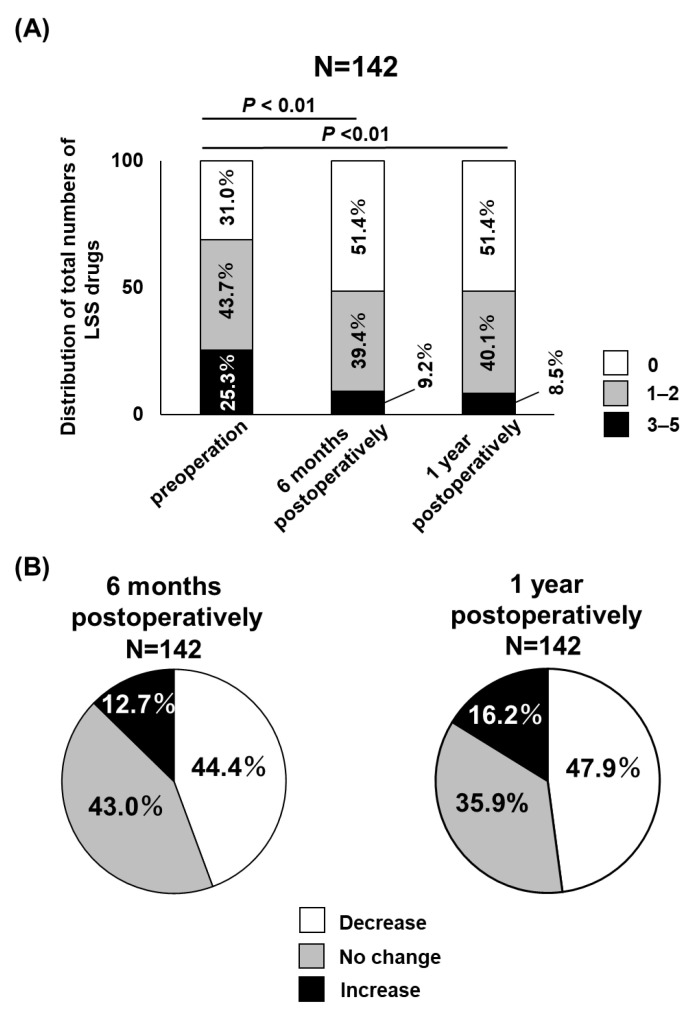
(**A**) Comparison of the preoperative and 6-month and 1-year postoperative number of LSCS medications in the participants. The proportion of patients taking from 3 to 5 LSCS medications gradually decreased after surgery. Conversely, the proportion of patients taking no LSCS medications gradually increased after surgery. There was a significant decrease in the proportion of patients taking LSCS medications, both 6 months and 1 year after surgery. (**B**) Distribution of changes in the number of LSCS medications 6 months (left panel) and 1 year after surgery (right panel).

First, the preoperative and 6-month and 1-year postoperative distribution of the number of LSCS medications in the participants were assessed (Figure 2A). The proportion of patients taking from three to five LSCS medications gradually decreased after surgery, and conversely, the proportion of patients taking no LSCS medications gradually increased after surgery (Figure 2A). Overall, there was a significant decrease in the proportion of patients taking LSCS medications, both 6 months and 1 year after surgery (Figure 2A). Figure 2B shows the distribution of changes in the number of LSCS medications 6 months and 1 year after surgery. A total of 44.4% showed a reduction in LSCS medications, 43.0% showed no change, and 12.7% showed an increase 6 months after surgery (Figure 2B). A total of 47.9% showed a reduction in LSCS medications, 35.9% showed no change, and 16.2% showed an increase 1 year after surgery (Figure 2B). The prevalence of an increase in LSCS medications 1 year after surgery (16.2%) was higher than that 6 months after surgery (12.7%) (Figure 2B). Next, we focused on the medications that were considered prescription medications for LSCS: NSAIDs, pregabalin/milogabalin, opioids, SNRIs, PGE1, acetaminophen, mecobalamin, and neurotropin. NSAIDs and pregabalin/milogabalin were the most frequently taken medications preoperatively (Table 3). The changes in the proportion of patients taking each LSCS medication before and after lumbar surgery were investigated. The proportion of patients taking NSAIDs, pregabalin/mirogabalin, opioids, PGE1, and neurotropin 6 months and 1 year after surgery was significantly lower than that before lumbar surgery (Table 3). Meanwhile, mecobalamin, acetaminophen, and SNRIs showed no significant change before and after lumbar surgery (Table 3).

**Figure 3:**
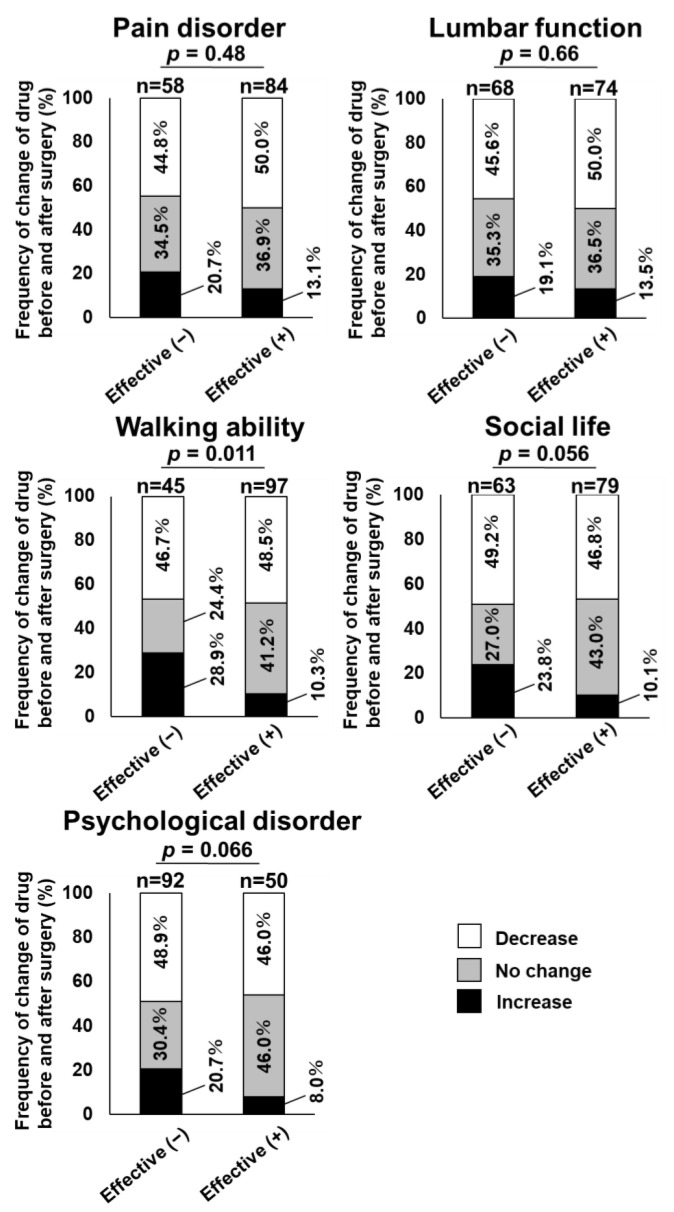
Comparison of the distribution of changes in the number of LSCS medications between effective and non-effective cases of surgical treatment in each domain of JOABPEQ. Walking ability showed a significant difference in the distribution of changes in the number of LSCS medications between the two groups. Social life showed a marginally significant difference, and the other three domains showed no significant difference between the two groups.

The distribution of changes in the number of LSCS medications was compared between effective and non-effective cases of surgical treatment in each domain of the JOABPEQ to clarify the relationship between surgical effectiveness after 1 year of surgery and postoperative changes in the number of LSCS medications (Figure 3). Walking ability showed a significant difference (*p* = 0.011) in the distribution of changes in the number of LSCS medications between the two groups. Meanwhile, social life showed a marginally significant difference (*p* = 0.056), and the other three domains showed no significant difference between the two groups. Next, the Poisson regression model was used to identify the factors associated with an increase in LSCS medications after 1 year of surgery. The multivariable analysis revealed that poor surgical effectiveness of walking ability (RR, 2.7; 95% CI: 1.3–5.9) and social life (RR, 2.3; 95% CI: 1.1–5.0) were significantly associated with an increase in LSCS medications after 1 year of surgery (Table 4). On the other hand, poor surgical effectiveness of pain disorder (RR, 1.3; 95% CI: 0.6–2.7), lumbar function (RR, 1.4; 95% CI: 0.7–2.9), and psychological disorder (RR, 2.3; 95% CI: 0.7–6.9) were not significantly associated with an increase in LSCS medications after 1 year of surgery (Table 4).

## 4. Discussion

This study clearly showed that the number of LSCS medications significantly decreased after lumbar surgery. The proportion of the patients taking NSAIDs, pregabalin/mirogabalin, opioids, PGE1, and neurotropin was significantly decreased after lumbar surgery, but that of the patients taking mecobalamin, acetaminophen, and SNRIs was not significantly changed. Additionally, the study results showed that around 15% of the participants showed an increase in LSCS medications even after lumbar surgery. The statistical analysis indicated that poor surgical effectiveness of walking ability and social life in JOABPEQ was significantly associated with an increase in LSCS medications 1 year after lumbar surgery.

Considering the effectiveness of lumbar surgery for patients with LSCS from various aspects including pain and motor function of lower extremities, social life, and psychological characteristics [12,13,14,15], the overall postoperative reduction in LSCS medications is reasonable and predictable. However, the findings that about 40% of patients had no change in the number of LSCS medications after surgery indicate that physicians may have aimlessly continued to prescribe their preoperative medications even after surgery. Given that most patients with LSCS are the elderly who must be aware of polypharmacy, which is deeply associated with the risk of adverse drug events (ADEs) [16], physicians should properly select LSCS medications for postoperative patients according to their symptoms. Meanwhile, around 15% of patients received more LSCS medications after surgery. When the postoperative increase in LSCS medications is considered one of the parameters of poor surgical outcomes, these results roughly agree with the finding that the frequency of poor surgical outcomes for patients with LSCS was 10%–20% in health-related quality of life (HRQOL) [17,18]. However, the change in the number of LSCS medications may not perfectly match that of HRQOL scores. In this study, the surgical effectiveness of pain disorder in JOABPEQ did not reflect the change in the number of LSCS medications. This result indicates that the use of medications in patients with LSCS does not depend on only pain in the lumbar and lower extremities. Meanwhile, the poor surgical outcomes of walking ability and social life in JOABPEQ were potentially involved in the postoperative increase in LSCS medications. Particularly, the poor surgical outcomes of walking ability in patients with LSCS were associated with increased LSCS medications. It is not entirely clear why the surgical effectiveness of walking ability is responsible for the change in the number of LSCS medications. However, these results indicate from another perspective that surgically indicated patients with LSCS may have the greatest expectation of improved walking ability in a variety of activities of daily living.

Among LSCS medications, NSAIDs and pregabalin/mirogabalin, which were more frequently taken preoperatively, were significantly less prescribed postoperatively. As NSAIDs require consideration for renal dysfunction and gastrointestinal disorders, short-term use or combined use of proton pump inhibitors is recommended according to the guideline [19,20,21]. NSAIDs are also considered one of the potentially inappropriate medications that can cause ADEs, especially for the elderly [19,20,21], so careful attention should be paid to the prescription of NSAIDs for LSCS patients, whether before or after lumbar surgery. Pregabalin/mirogabalin has been also reported to have a relatively high incidence of ADEs in patients with LSCS [22,23]. Common ADEs associated with pregabalin and mirogabalin are somnolence, dizziness, peripheral edema, and weight gain [22,23]. Especially in elderly patients with LSCS, physicians need to be aware of somnolence and dizziness caused by pregabalin/mirogabalin in terms of increased risk of falls. Meanwhile, mecobalamin and acetaminophen had a relatively high frequency of use and no significant change in frequency before and after surgery. These medications tend to be prescribed continuously for residual pain and numbness after surgery because they have fewer ADEs, unlike NSAIDs and pregabalin/mirogabalin [6,24]. In the present study, the frequency of patients taking PGE1 was not high compared with NSAIDs and pregabalin/mirogabalin. According to the guideline [1], although PGE1 has no evidence of efficacy against the nerve root type of LSCS, it has clear evidence of efficacy for patients with the cauda equina type or combined type of LSCS. In addition, PGE1 is considered a highly safe medication for LSCS patients compared with NSAIDs and pregabalin/mirogabalin [1]. Taken together, the study results indicate that lumbar surgery has the potential to reduce the risk of ADEs of LSCS medications. Meanwhile, lumbar surgery was reported to have a reoperation rate of 8% [25]. In addition, Ma et al. reported that the incidence of complications caused by lumbar surgery was around 20% [26]. Therefore, although it is necessary to consider reducing the risk of ADEs with LSCS medications, physicians must switch from conservative therapy to lumbar surgery for LSCS patients with adequate consideration for the possibility of adverse events caused by lumbar surgery.

This study has several limitations. First, the data used in this study were collected from a single institution. Because the LSCS medications were prescribed at the discretion of the individual physician in this study, these results may reflect physicians’ preferences or patients’ pressures. The study results are based on the patients seen by six spinal surgeons but should be validated by the data derived from more physicians at multiple institutions. Second, the follow-up duration of this study was 1 year. A longer follow-up period is necessary because the patient’s symptoms and the LSCS medications may change more than 1 year after lumbar surgery. Third, although treatments other than surgical therapy can also affect the medications of patients with LSCS, we did not assess therapies other than lumbar surgery and pharmacological treatment in this study. In the future, we should consider not only the effectiveness of surgical treatment but also the status of the other conservative treatments, including physical therapy, exercise, and spinal injections, to assess the change of medications in patients with LSCS. Fourth, the study covered only medications prescribed in hospitals or clinics. In this study, we did not consider that patients might use medications over the counter. This variable may have influenced the results. Lastly, this study did not assess the costs incurred over the course of 1 year for these cohorts. From a medical economic perspective, the total cost of treatment, including conservative therapy and lumbar surgery, should be compared among surgical patients with LSCS. In the future, the best treatment strategy for LSCS must also be established in terms of cost-effectiveness. However, to the best of our knowledge, this is the first follow-up study to assess the LSCS medications of lumbar surgical patients. The study results may provide physicians with beneficial information on treatment for LSCS.

## 5. Conclusions

This study demonstrated the impact of lumbar surgery on pharmacological treatment for patients with LSCS. About 15% of the participants showed an increase in LSCS medications 1 year after lumbar surgery. The multivariable analysis revealed that individuals without improvements in walking ability or social life had a greater risk of a postoperative increase in LSCS medications.

## Figures and Tables

**Table 1:** Baseline characteristics of participants.

Patients		*n* = 142
Gender		Male: 84 Female: 58
Age (years)		70.1 ± 10.3
BMI (kg/m^2^)		24.1 ± 3.4
Medical history	Diabetes mellitus	38 (26.8%)
	Hypertension	76 (53.5%)
	Dyslipidemia	61 (43.0%)
	Cardiovascular disease	42 (29.6%)
	Cerebrovascular disease	12 (8.5%)
	Cancer	21 (14.8%)
Spondylolisthesis		50 (35.2%)
Degenerative lumbar scoliosis		16 (11.2%)
FBSS		11 (7.7%)
Surgical procedure	decompression	84 (59.2%)
	decompression + fusion	58 (40.8%)
Surgical time (min)		139.8 ± 90.3
Surgical blood loss (mL)		149.5 ± 169.8
Incidental dural tear		13 (9.2%)

BMI, Body mass index; FBSS, failed back surgery syndrome.

**Table 2:** The number of effective cases of surgical treatment on JOA Back Pain Evaluation Questionnaire (JOABPEQ) (*n* = 142).

	6POM	1POY
Pain disorder	92 (64.1%)	84 (59.1%)
Lumbar function	75 (52.8%)	74 (52.1%)
Walking ability	99 (69.7%)	97 (68.3%)
Social life	68 (47.9%)	79 (55.6%)
Psychological disorder	43 (30.3%)	50 (35.2%)

6POM, 6 postoperative months; 1POY, 1 postoperative year.

**Table 3:** Proportion of patients taking each LSCS medication before and after surgery (*n* = 142).

	Preoperation	6POM	1POY	*p* Value *	
				Preoperation vs. 6POM	Preoperation vs. 1POY
NSAIDs	53 (37.3%)	39 (27.5%)	33 (23.2%)	0.03	<0.01
pregabalin/mirogabalin	51 (35.9%)	29 (20.4%)	30 (21.1%)	<0.01	<0.01
mecobalamin	30 (21.1%)	23 (16.2%)	26 (18.3%)	0.09	0.35
opioids	24 (16.9%)	8 (5.6%)	12 (8.5%)	<0.01	<0.01
PGE1	20 (14.1%)	6 (4.2%)	6 (4.2%)	<0.01	<0.01
acetaminophen	14 (9.9%)	12 (8.5%)	13 (9.2%)	0.53	0.76
SNRIs	10 (7.0%)	8 (5.6%)	6 (4.2%)	0.48	0.16
neurotropin	8 (5.6%)	1 (0.7%)	1 (0.7%)	0.02	0.02

NSAIDs, non-steroidal anti-inflammatory drugs; PGE1, prostaglandin E1 analogs; SNRIs, serotonin-noradrenalin reuptake inhibitors; 6POM, 6 postoperative months; 1POY, 1 postoperative year. * Wilcoxon signed-rank test.

**Table 4:** Poisson regression model for increase of LSCS medications after 1 year of surgery.

	Total Number	Number of Case	Prevalence of Case	Relative Risk *	95% Confidence Interval	*p*-Value
Pain disorder						
Effective (+)	84	11	13.1	Reference		
Effective (−)	58	12	20.7	1.3	(0.6–2.7)	0.49
Lumbar function						
Effective (+)	74	10	13.5	Reference		
Effective (−)	68	13	19.1	1.4	(0.7–2.9)	0.34
Walking ability						
Effective (+)	97	10	10.3	Reference		
Effective (−)	45	13	28.9	2.7	(1.3–5.9)	0.01
Social life						
Effective (+)	79	8	10.1	Reference		
Effective (−)	63	15	23.8	2.3	(1.1–5.0)	0.03
Psychological disorder						
Effective (+)	50	4	8.0	Reference		
Effective (−)	92	19	20.7	2.3	(0.7–6.9)	0.15

* Adjusted for age, sex, body mass index, history of diabetes, with or without fixation, and incidental dural injury.

## Data Availability

The datasets generated and analyzed during this study are not publicly available due to limitations of ethical approval involving the patient data and anonymity but are available from the corresponding author upon reasonable request.
